# Tele-rehabilitation for Type II diabetics with heart failure with preserved ejection fraction

**DOI:** 10.3389/fendo.2024.1433297

**Published:** 2024-07-02

**Authors:** Minjie Yuan, Haimin Xu, Dongqi Zhao, Dongdong Shi, Li Su, Huifang Zhu, Shengdi Lu, Junbo Wei

**Affiliations:** ^1^ Department of Cardiology, Renhe Hospital, Baoshan District, Shanghai, China; ^2^ Department of Orthopedics, Shanghai Sixth People’s Hospital Affiliated to Shanghai Jiao Tong University School of Medicine, Shanghai, China

**Keywords:** tele-rehabilitation, diabetes, HFPEF, Propensity score matching, outcome

## Abstract

**Objective:**

This study aims to determine whether tele-rehabilitation has similar effects to conventional face-to-face physical rehabilitation for diabetic patients with heart failure with preserved ejection fraction (HFpEF).

**Materials and methods:**

Demographic, laboratory, diagnostic and rehabilitation information for patients with type 2 diabetes with HFpEF were extracted from disease-specific databases. Outcome measures, including the Short Physical Performance Battery (SPPB), 6-minute walk distance, frailty status, European Quality of Life 5-Dimension 5-Level questionnaire (EQ-5D-5L) and reduction in HbA1c from admission, patients who received tele-rehabilitation therapy were compared to those received face-to-face rehabilitation.

**Results:**

In this study, 90 patients with type 2 diabetes and HFpEF using tele-rehabilitation were matched with 90 patients with type 2 diabetes and HFpEF using face-to-face physical rehabilitation. Improvements in the results of the SPPB scores, 6-min walk distance and gait speed and EQ-5D-5L were noted from the follow-up time point 3 months to 6 months in both two groups. There were no significant differences in functional tests and quality of life between the two groups.

**Conclusion:**

Our study proved that mobile-based tele-rehabilitation programs are non-inferior to face-to-face physical rehabilitation for diabetes patients after HFpEF. In addition, adherence to the telerehabilitation program showed that the novel technology was accepted well and could be an alternative to the conventional face-to-face rehabilitation program.

## Introduction

Heart failure, a prevalent condition among elderly individuals globally, stands as a primary reason for hospital admissions. It correlates with diminished health-related quality of life, frequent readmissions to hospitals, and significant mortality rates (1, 2). There are a number of reasons why a person with diabetes may be at risk for heart failure: 1) Chronic high blood sugar, one of its main features, damages blood vessels and the nerves that control the heart. In the long run, these damages may cause cardiovascular diseases including heart failure. High glucose levels can lead to the formation of plaques in the arteries (atherosclerosis), reducing blood flow and increasing the risk of heart attacks (3). 2) Insulin resistance in type 2 diabetes mellitus (4). Insulin is a hormone that helps regulate blood glucose levels. Insulin resistance not only contributes to hyperglycemia but is also associated with a cluster of cardiovascular risk factors, including hypertension, abnormal cholesterol levels, and obesity. These conditions, often called “metabolic syndrome,” can greatly increase the risk of heart disease and heart failure. 3) Diabetes is accompanied by a higher inflammatory response and more oxidative stress, which can lead to damage to the cardiovascular system. Inflammatory cytokines and oxidative stress can lead to endothelial dysfunction, a condition in which the inner lining of blood vessels does not function normally, further increasing the risk of cardiovascular disease. 4) People with diabetes often have hypertension, which can put extra strain on the heart, leading to heart muscle thickening (hypertrophy) and eventually heart failure.

In patients with heart failure, there is a significant decline in body function and a high incidence of debilitation, which is exacerbated by the presence of concurrent diseases (5–8). Even in elderly patients with stabilized heart failure who are well cared for, body functions are often severely impaired by aging, cardiovascular dysfunction, and musculoskeletal dysfunction (9, 10). In patients with heart failure who convert to acute decompensated heart failure, the organism functions worse and can be exacerbated by hospital admissions and bed rest (8). The deficits are usually long-lasting. Many patients fail to return to basic functioning, have reduced self-care, and are at higher risk for readmission and death after discharge.

The Rehabilitation Therapy in Older Acute Heart Failure Patients (REHAB-HF) trial was a multicenter, single-blinded, randomized controlled trial, which focused on an early, personalized, progressive rehabilitation intervention encompassing various aspects of physical function. The study demonstrated that for older adults admitted to the hospital due to acute decompensated heart failure, receiving transitional, tailored and progressive rehabilitation interventions (covering various aspects of physical function) for 12 weeks after admission resulted in significant improvements in physical function compared to usual care (11).

Due in part to the trend in the aging of the populations in big cities in China, which result in a high prevalence of heart failure, the number of patients has steadily increased over the past decades while hospital stays have decreased. This patient group has consequently become a growing workload for physical therapists (PT) either in the community or hospital. In some communities in the author’s home city (Shanghai, China), these patients group approximately accounts for over 30% of PT’s caseload, and this number is increasing year by year. Since the growing rehabilitation needs of heart failure cannot be met by the current labor force of PT in China, the exploration for new effective alternatives to ensure reliable and accessible postoperative physical rehabilitation is vital and urgent (12).

Another treatment model is to use telerehabilitation technology to deliver rehabilitation programs directly to the patient’s home. This intervention may mitigate access challenges for patients residing in both rural and remote areas, as well as those in urban settings facing transportation difficulties (13, 14). Many patients might encounter difficulties accessing healthcare services following hospital discharge. Access to rehabilitation programs is complex with patients’ financial costs and health sectors providing in-home services in conjunction with or substitute with community care. For patients living in rural areas, the problem of access is compounded by long distances and time spent by patients or treating doctors. Home-based programs facilitated by technology might motivate patients to engage in more frequent physical activity, which could help mitigate the strength deficits commonly observed in older individuals with diabetes and heart failure. It not only provides convenience for patients, but also saves healthcare costs.

This study aims to determine whether tele-rehabilitation has similar effects to conventional face-to-face physical rehabilitation for diabetic patients with HFpEF.

## Materials and methods

### Study participants

Data on patients with HFpEF were obtained in “Heart Failure database”, which is one of the disease-specific databases in Renhe Hospital, Baoshan District, Shanghai. The Heart Failure database included electronic health record data from March 1, 2018. For the present study, data from the Heart Failure database were included in the analysis. Full details of the inclusion and exclusion criteria of the data base are provided in the **Supplementary Appendix S1**.

The definition of type 2 diabetes in the present study was formulated according to the SUPREME-DM criteria (15) as follows: a) One or more International Classification of Diseases, Ninth Revision, Clinical Modification (ICD-9-CM) codes and Tenth Revision, Clinical Modification (ICD-10-CM) codes for type 2 diabetes associated with an inpatient hospitalization; b) Two or more ICD codes associated with outpatient visits on different dates within a 2-year period; c) Two or more codes associated with outpatient visits on different dates within a 2-year period. combinations of: 1) ICD codes associated with outpatient visits; 2) fasting glucose levels ≥ 126 mg/dl; 3) 2-hour glucose level ≥ 200 mg/dl; 4) random glucose ≥ 200 mg/dl; 5) HbA1c ≥ 6.5%; and 6) prescriptions for antidiabetic medications.

Although different cut points have been defined in the literature (16), and EF <50% could be sub-divided into midrange (40% to 49%) and reduced (<40%) classifications (17). We defined HFpEF by an EF ≥ 50%, in consistency with previous analyses of hospital readmissions in patients discharged with heart failure.

**Figure 1:**
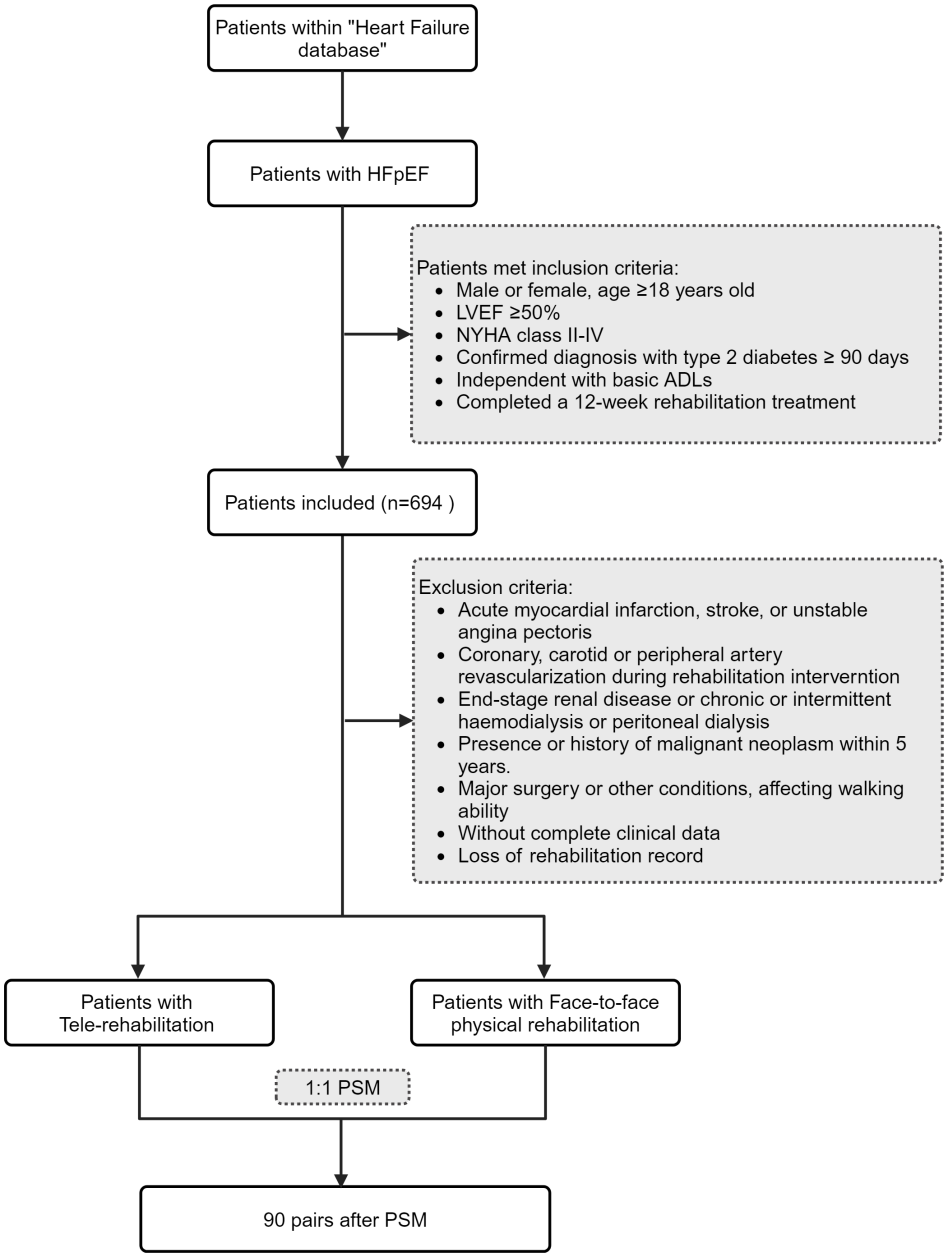
Flowchart of the study.

A total of 694 patients with type 2 diabetes mellitus combined with HFpEF were identified in this study. After excluding patients with incomplete data, a total of 576 patients with type 2 diabetes mellitus combined with HFpEF were included in this study (**Figure 1**). The study and analysis plan were approved by the Institutional Review Board (Research Ethics Committee) of Renhe Hospital, Baoshan District, Shanghai, China (KY2023–18). As we used anonymized data from electronic medical records, informed consent was not obtained from the participating researchers.

### Interventions

#### Face-to-face physical rehabilitation

If possible, intervention is provided in the hospital and then transferred to an outpatient facility as soon as possible after discharge. If necessary, interventionists will provide in-home treatment until the patient is physically able to participate in outpatient treatment, if the patient is physically able to do so. Outpatient therapy lasted 60 minutes, 3 days per week, for 12 weeks (or 36 sessions), with an interventionist-to-patient ratio of 1:1. Outpatient therapy was accompanied by home exercise on non-treatment days (low-intensity walking gradually increased to 30 to 45 minutes per day and rehabilitation exercises). Each session included approximately 15 to 20 minutes of rehabilitation exercises including four domains tailored to the patient’s condition. For patients in the face-to-face physical rehabilitation group, exercises were conducted at the outpatient facility with one-on-one guidance from a PT. For patients in the tele-rehabilitation group, exercises were conducted at home, monitored via an APP and sensors. Every two weeks, patients were reassessed at the PT outpatient clinic to determine if their exercise prescriptions needed adjustments. For the tele-rehabilitation group, these assessments were conducted by the PT through real-time bidirectional video and audio interactions online. Detailed information was provided in **Supplementary Appendix S2**. The home exercise component of the intervention was initiated after the home environment was evaluated through remote video assessment by a PT and deemed suitable for rehabilitation exercises (11).

The primary purpose of the first 3 months (outpatient phase) of the intervention is to prepare patients for the transition to the independent maintenance phase (months 4 to 6). At the 3-month visit, patients are provided with an individualized exercise prescription and then followed up every 4 weeks via telephone contact. Retention rates and adherence to the intervention sessions for patients in the intervention group are reviewed and discussed every 2 weeks by a special committee, as recommended by the National Institutes of Health Behaviour Change Consortium Treatment Fidelity Workgroup (18).

#### Tele-rehabilitation

Remote rehabilitation interventions were carried out through the mobile application Joymotion^®^ software (developed by Shanghai Medmotion Medical Management Co., Ltd., Shanghai, China). This application facilitated the provision of exercise guidelines, feedback on training performance, and enabled real-time bidirectional video and audio interactions between patients and PTs (19).

Originally developed for the rehabilitation of musculoskeletal disorders, the Joymotion^®^ APP has expanded its scope through advancements in telemedicine, remote sensing rehabilitation technologies, and the accumulation of a diverse exercise database. Due to the substantial overlap between the rehabilitation needs of patients with heart failure, metabolic diseases, and those with musculoskeletal disorders, this APP has been co-developed and upgraded in collaboration with cardiology and orthopedic departments to tailor rehabilitation programs specifically for cardiac and metabolic conditions. The APP, installed by a technician on the day of the patient’s discharge, connected through the patient’s home Wi-Fi. PTs at the rehabilitation center would initiate weekly scheduled teleconference sessions with the patients. The APP provided daily rehabilitation exercises with comprehensive instructions and tracked exercise completion rates. The rehabilitation protocol, prescribed by the supervising PT, was conveyed to patients as “daily tasks”. The intervention content mirrored that of the face-to-face group. Adherence to the prescribed regimen and retention of patients within the intervention group were monitored through the APP by the supervising PTs, with the completion of daily tasks being trackable via the APP’s website.

#### Baseline measurement

Patients’ data extracted from the Heart Failure database for this study included birth date, sex, body mass index (BMI), insurance type, date of diagnosis of heart failure, date of diagnosis of diabetes mellitus, blood pressure, smoking status, diagnosis of various medical conditions, laboratory tests, ejection fraction from cardiac ultrasonography, NYHA (New York Heart Association) class, median N-terminal pro–B-type natriuretic peptide. Based on smoking status reported at each clinical visit, we categorized patients into three groups: never smokers, former smokers, and current smokers.

Tele-rehabilitation interventions started on 15 January 2021 at Renhe Hospital, Baoshan District, Shanghai. Tele-rehabilitation users (Group TELE) were defined as patients who used tele-rehabilitation immediately after discharge from hospital, and no face-to-face physical rehabilitation visit was recorded. Non-users of tele-rehabilitation (Group PT) were defined as patients who regularly visited physical rehabilitation departments or clinics 3–4 times per week as required by doctors.

#### Propensity score matching (PSM)

Patients included in this study were required to have a follow-up period of no less than six months post-hospital discharge. Initially, patients with type 2 diabetes and HFpEF who received tele-rehabilitation were identified. Propensity score matching was performed using the nearest neighbor algorithm, with a caliper set at 0.01. Matching covariates included age, sex, baseline BMI, systolic blood pressure, smoking status, insurance type, NYHA (New York Heart Association) class, and median N-terminal pro-B-type natriuretic peptide levels. Based on these propensity scores, patients undergoing traditional physical rehabilitation were matched in a 1:1 ratio with those receiving tele-rehabilitation interventions (**Figure 1**).

Ultimately, 90 patients with type 2 diabetes and HFpEF who utilized tele-rehabilitation were matched with 90 patients who underwent in-person physical rehabilitation, resulting in a total cohort of 180 patients. The baseline characteristics between the groups were well balanced (**Table 1**).

**Table 1:** Demographic and clinical characteristics of the patients at baseline*.

Characteristic	Total (n=180)	Group PT (n=90)	Group TELE (n=90)	P- value
Age -yr, mean (SD)	64.04 (10.60)	63.43 (11.15)	64.64 (10.05)	0.445
Female sex — no (%)	74 (41.11)	38 (42.22)	36 (40.00)	0.880
Body mass index -kg/m^2^, mean (SD)	24.28 (3.67)	24.26 (3.53)	24.31 (3.83)	0.934
NYHA class — no (%)				0.629
II	33 (18.33)	19 (21.11)	14 (15.56)	
III	114 (63.33)	55 (61.11)	59 (65.56)	
IV	33 (18.33)	16 (17.78)	17 (18.89)	
HbA1c at admission (%)	7.46 (1.67)	7.37 (1.62)	7.56 (1.71)	0.446
Median N-terminal pro–B-type natriuretic peptide -pg/ml, median [IQR]	636.75 [561.16, 953.20]	613.00 [559.23, 792.20]	677.55 [570.31, 1134.20]	0.208
Smoking status— no (%)				0.971
Never	95 (52.78)	47 (52.22)	48 (53.33)	
Ever	64 (35.56)	32 (35.56)	32 (35.56)	
Current	21 (11.67)	11 (12.22)	10 (11.11)	
Insurance — no (%)				0.463
None	21 (11.67)	13 (14.44)	8 (8.89)	
Social	145 (80.56)	71 (78.89)	74 (82.22)	
Commercial	14 (7.78)	6 (6.67)	8 (8.89)	
Coexisting conditions				
Total no. of coexisting conditions	2.64 (0.98)	2.67 (0.89)	2.62 (1.08)	0.763
Hypertension — no (%)	167 (92.78)	83 (92.22)	84 (93.33)	1.000
History of myocardial infarction — no (%)	17 (9.44)	7 (7.78)	10 (11.11)	0.606
History of coronary revascularization, including PCI and CABG — no (%)	36 (20.00)	17 (18.89)	19 (21.11)	0.845
Atrial fibrillation — no (%)	92 (51.11)	51 (56.67)	41 (45.56)	0.185
Hyperlipidemia — no (%)	133 (73.89)	67 (74.44)	66 (73.33)	1.000
Depression, according to electronic medical record — no (%)	31 (17.22)	15 (16.67)	16 (17.78)	1.000
Geriatric conditions				
Dementia or cognitive impairment, according to electronic medical record — no (%)	6 (3.33)	1 (1.11)	5 (5.56)	0.221
Frail, as defined by the presence of at least three Fried criteria† — no (%)	84 (46.67)	40 (44.44)	44 (48.89)	0.658
Prefrail, as defined by the presence of one or two Fried criteria† — no (%)	96 (53.33)	50 (55.56)	46 (51.11)	0.658
Urinary incontinence — no (%)	23 (12.78)	8 (8.89)	15 (16.67)	0.211

Continuous variables were expressed as mean ± standard deviation (SD), or median [interquartile range] and were compared utilizing either independent t test or Mann-Whitney U test. Categorical variables were presented as the number of cases (percentage) and were compared utilizing Chi-square test.

*CABG denotes coronary artery bypass graft, IQR interquartile range, NYHA New York Heart Association, and PCI percutaneous coronary intervention.

†The five Fried criteria include weight loss, exhaustion, low physical activity, slow gait speed, and weak hand-grip strength.

### Outcomes measures

Patients’ outcome data at day of discharge (baseline), 3 months (3 mo) and 6 months (6 mo) after discharge were extracted, including score on the SPPB, 6-min walk distance, frailty status [assessed according to modified Fried criteria (5)], and EQ-5D-5L visual-analogue scale, and decreased HbA1c from admission.

The primary outcome of this study was SPPB score, a standardized and reproducible measure of overall physical function. The SPPB has been validated in frail older adults and is known to predict a broad spectrum of clinical outcomes (20–22).

### Statistical analysis

The groups were first compared on baseline characteristics. Continuous variables were expressed as mean ± standard deviation (SD) or median (interquartile range) and were compared utilizing either the paired t test or the Wilcoxon sign rank test, depending on the Shapiro-Wilk test. The categorical variables were presented as the number of cases (percentage) and statistically analyzed with McNemar or McNemar-Bowker test. The main research hypothesis verified that the mean SPPB score gain from baseline to the last follow-up in the Tele-rehabilitation group would not be inferior compared with that in the control group. Only patients who participated in all assessments and attended the required number of intervention sessions, as specified in the inclusion criteria in S1, were included in the analysis. All statistical analyses were performed by SPSS 27.0 with a significance level of 0.05 (two-sided).

## Results

In this study, 90 patients with type 2 diabetes mellitus combined with HFpEF using telerehabilitation and 90 patients using face-to-face physical rehabilitation were matched, totaling 180. The baseline characteristics of the two groups were well matched (**Table 1**).

For primary outcome and major secondary outcome, improvements in the results of the SPPB scores, 6-min walk distance and gait speed were noted from the follow-up time point 3 months to 6 months in both two groups, patients using tele-rehabilitation had similar SPPB scores and 6-min walk distance (and gait speed) with patients using face-to-face physical rehabilitation at both 3 months and 6 months after discharge. The gains from 3 months to 6 months showed no significant differences with regards to SPPB scores and 6-min walk distance (and gait speed) (**Table 2**).

**Table 2:** Heart failure and diabetes mellitus related outcomes.

Outcomes	Group PT (n=90)	Group TELE (n=90)	P-value
SPPB score, primary outcome†			
At baseline	5.30 (1.62)	5.26 (1.55)	0.860
At 3 mo	8.09 (0.79)	8.12 (0.72)	0.781
At 6 mo	9.21 (0.61)	9.21 (0.66)	1.000
Change from baseline			
To 3 mo	2.79 (1.53)	2.87 (1.52)	0.745
To 6 mo	3.91 (1.63)	3.96 (1.56)	0.862
Balance score			
At baseline	1.92 (0.88)	1.89 (0.90)	0.805
At 3 mo	2.81 (0.58)	2.89 (0.59)	0.409
At 6 mo	3.10 (0.37)	3.09 (0.36)	0.820
Change from baseline			
To 3 mo	0.89 (0.64)	1.00 (0.73)	0.283
To 6 mo	1.18 (0.91)	1.20 (0.93)	0.870
4-M walk score			
At baseline	1.91 (0.82)	1.98 (0.83)	0.574
At 3 mo	3.26 (0.49)	3.22 (0.51)	0.671
At 6 mo	3.90 (0.30)	3.87 (0.34)	0.470
Change from baseline			
To 3 mo	1.34 (0.81)	1.24 (0.96)	0.424
To 6 mo	1.99 (0.83)	1.89 (0.74)	0.378
Chair rise score			
At baseline	1.47 (0.97)	1.39 (0.94)	0.621
At 3 mo	2.02 (0.15)	2.01 (0.11)	0.567
At 6 mo	2.21 (0.41)	2.26 (0.44)	0.530
Change from baseline			
To 3 mo	0.56 (1.01)	0.62 (0.95)	0.681
To 6 mo	0.74 (1.00)	0.87 (1.02)	0.470
6-min walk distance — m			
At baseline	193.00 (54.66)	197.53 (52.95)	0.555
At 3 mo	285.43 (27.23)	279.69 (29.87)	0.187
At 6 mo	327.70 (20.50)	323.56 (18.85)	0.148
Change from baseline			
To 3 mo	92.43 (54.42)	82.16 (57.84)	0.165
To 6 mo	134.70 (55.39)	126.02 (48.52)	0.248
Frailty status — no. of modified Fried criteria met‡			
At baseline	2.42 (0.81)	2.47 (0.81)	0.710
At 3 mo	1.67 (0.52)	1.62 (0.57)	0.558
At 6 mo	0.86 (0.46)	0.83 (0.50)	0.734
Change from baseline			
To 3 mo	-0.76 (0.77)	-0.84 (0.73)	0.417
To 6 mo	-1.57 (0.90)	-1.63 (0.76)	0.602
EQ-5D-5L visual-analogue scale score ††			
At baseline	0.52 (0.10)	0.51 (0.09)	0.678
At 3 mo	0.64 (0.07)	0.65 (0.05)	0.145
At 6 mo	0.72 (0.05)	0.72 (0.04)	0.968
Change from baseline			
To 3 mo	0.12 (0.08)	0.14 (0.10)	0.162
To 6 mo	0.20 (0.10)	0.21 (0.09)	0.719
Decreased HbA1c from admission — % †††			
At 3 mo	-0.71 (0.18)	-0.70 (0.22)	0.951
At 6 mo	-1.01 (0.25)	-1.04 (0.24)	0.367

Continuous variables were expressed as mean ± standard deviation (SD) and were compared utilizing paired t test.

† Total scores on the Short Physical Performance Battery (SPPB) range from 0 to 12, with lower scores indicating more severe physical dysfunction; each component (the standing balance test, the gait-speed test [as assessed by a 4-m walk], and the strength test [as assessed by the time needed to rise from a chair five times]) is scored on a scale of 0 to 4.

‡ Frailty status was assessed according to modified Fried criteria^5^.

†† Scores on the European Quality of Life 5-Dimension 5-Level questionnaire (EQ-5D-5L) visual analogue scale range from 0 to 1, with higher scores indicating better health status.

††† Decreased HbA1c are presented as HbA1c at 3 mo or 6 mo minus HbA1c at admission.

The other secondary outcome, frailty status, quality of life and clinical events also improved from 3 months to 6 months, patients using tele-rehabilitation noted similar EQ-5D-5L scores with patients using face-to-face physical rehabilitation at 6 months (**Table 2**). Decreased HbA1c from baseline were similar for two groups at both 3 months and 6 months after discharge.

For clinical events, the number of events was similar among two groups, and the number of days of rehospitalization for any cause also noted no significant difference (**Table 3**).

**Table 3:** Clinical events.

Clinical events at 6 mo	Group PT (n=90)	Group TELE (n=90)	P-value
Rehospitalization for any cause, secondary outcome — no. of events (rate)	24 (0.27)	18 (0.20)	0.400
Death — no. of events (rate)	0	0	1.000
Rehospitalization for heart failure — no. of events (rate)	6 (0.07)	7 (0.08)	1.000
No. of patients with ≥2 rehospitalizations for any cause (%)	4 (4.44)	3 (3.33)	1.000
No. of patients with ≥2 rehospitalizations for heart failure (%)	2 (2.22)	2 (2.22)	1.000
No. of days of rehospitalization for any cause	1.11 (2.20)	0.94 (2.55)	0.639
No. of patients with ≥1 fall (%)	24 (26.67)	19 (21.11)	0.484
No. of patients with ≥1 fall that resulted in injury (%)	1 (1.11)	3 (3.33)	0.613

## Discussion

The “Heart Failure database”, founded on March 1, 2018, is one of the disease-specific databases in Renhe Hospital, Baoshan District, Shanghai. The inclusion criteria were provided in the **Supplementary Appendix S1**. Using real-world disease-specific database information, this study found that patients with type 2 diabetes mellitus combined with HFpEF using tele-rehabilitation therapy was comparable to the efficacy of face-to-face physical rehabilitation. Studies in terms of debilitating conditions and quality of survival also demonstrated the clinical effectiveness of tele-rehabilitation.

Our trial sought to fill significant evidence gaps in tele-rehabilitation for patients with diabetes with heart failure. Unlike previous early heart failure rehabilitation trials, which typically started patient enrollment and intervention about seven weeks post-discharge and primarily used traditional endurance exercise training (23, 24), our trial began earlier and included a more diverse and frail patient population (24). For instance, the EJECTION-HF trial (Exercise Joins Education: Combined Therapy to Improve Outcomes in Newly-Discharged Heart Failure) found no advantage of the intervention over usual care regarding 6-minute walk distance, rehospitalization, and mortality, with adherence at only 43% (23, 24). In contrast, the REHAB-HF trial, focusing on frail, older patients hospitalized for acute decompensated heart failure, showed that an early, tailored, and progressive rehabilitation approach led to significantly better physical function outcomes than usual care (11).

Tele-rehabilitation is widely used for glycemic control, exercise capacity, physical fitness, muscle strength and psychosocial status in people with type 2 diabetes mellitus (25). Tele-rehabilitation was commercially available in Renhe Hospital, Baoshan District, Shanghai on 15 January 2021 and was applied to patients with heart failure after discharge. To our knowledge, our analyses were the first study to evaluate the clinical effects of tele-rehabilitation in diabetes with heart failure with preserved EF, and the results supported our hypothesis of non-inferiority of tele-rehabilitation in function, frailty status and quality of life compared with traditional physical rehabilitation. The data in this study provide an essential reference for a more comprehensive and in-depth study on the effectiveness of telerehabilitation in different countries and regions, especially in the fields of rehabilitation after heart failure.

Several limitations in the current study are acknowledged. Firstly, the sample size of patients participating in tele-rehabilitation in our study was relatively small compared to other real-world studies. Secondly, the PSM process was not optimal for certain covariates, as some socioeconomic variables, such as education level and family income, were absent in the electronic medical records (EMR) data. Thirdly, the long-term effects of this rehabilitation program are unknown, the limited follow-up period of 6 months has implications for the interpretation of the results. Further randomized controlled trials with larger samples are needed to evaluate telerehabilitation in patients after acute heart failure. Lastly, the inherent limitations of a single-center study particularly restricted the generalizability. Differences in medical standards, economic levels, infrastructure, and patients’ awareness of rehabilitation treatments across different cities can potentially impact rehabilitation outcomes. Our study is an initial step in establishing the feasibility and effectiveness of tele-rehabilitation. We hope that future researchers will consider conducting multi-center studies that encompass multiple cities and hospitals in subsequent clinical trials.

## Conclusion

Our study proved that mobile-based tele-rehabilitation programs are non-inferior to face-to-face physical rehabilitation for diabetes patients after acute heart failure. In addition, adherence to the telerehabilitation program showed that the novel technology was accepted well and could be an alternative to the conventional face-to-face rehabilitation program.

## Data availability statement

The raw data supporting the conclusions of this article will be made available by the authors, without undue reservation.

## Ethics statement

The studies involving humans were approved by Institutional Review Board (Research Ethics Committee) of Renhe Hospital, Baoshan District, Shanghai. The studies were conducted in accordance with the local legislation and institutional requirements. The ethics committee/institutional review board waived the requirement of written informed consent for participation from the participants or the participants’ legal guardians/next of kin because this study is a retrospective study, and there is no identifiable patient information involved.

## Author contributions

MY: Formal Analysis, Methodology, Software, Writing – original draft. HX: Data curation, Funding acquisition, Methodology, Writing – original draft. DZ: Investigation, Methodology, Writing – original draft. DS: Software, Writing – original draft. LS: Data curation, Writing – original draft. HZ: Methodology, Writing – original draft. SL: Supervision, Validation, Writing – review & editing. JW: Conceptualization, Funding acquisition, Project administration, Writing – review & editing.
