# Supplementary material for: Tele-rehabilitation for Type II diabetics with heart failure with preserved ejection fraction
Source: Front Endocrinol (Lausanne). 2024 Jul 2;15:1433297. doi: 10.3389/fendo.2024.1433297 (PMC11250425; doi:10.3389/fendo.2024.1433297)
Supplement: Supplementary file 1 [file DataSheet_1.docx]

S1. inclusion and exclusion criteria

**Inclusion criteria**

- Male or female, age ≥18 years old
- LVEF ≥50%
- NYHA class II-IV
- Confirmed diagnosis with type 2 diabetes ≥ 90 days
- Diagnosed with HFpEF which required at least one of the following: elevated estimated LV filling pressure [E/é medial ≥15] or E/é medial of 8 or greater with concurrent elevated natriuretic peptides [NT-proBNP ≥220 pg/mL or BNP ≥80 pg/mL]
- Patient was independent with basic activities of daily living (ADLs) including the ability to ambulate independently (with or without the use of an assistive device)
- Patients completed a 12-week rehabilitation treatment, by remote resistance exercises programs or attending the clinic for follow-up visits at a frequency of no less than once every week, and availability of follow-up data at the 3-month and 6-month intervals.

**Exclusion criteria**

- Acute myocardial infarction, stroke, or unstable angina pectoris
- Coronary, carotid or peripheral artery revascularization during rehabilitation interverntion
- End-stage renal disease or chronic or intermittent haemodialysis or peritoneal dialysis
- Presence or history of malignant neoplasm within 5 years. Basal and squamous cell cancer and any carcinoma in-situ are allowed
- Major surgery or other conditions, affecting walking ability
- Patients without complete clinical data
- Patients with loss of rehabilitation record
