# Supplementary material for: Tele-rehabilitation for Type II diabetics with heart failure with preserved ejection fraction
Source: Front Endocrinol (Lausanne). 2024 Jul 2;15:1433297. doi: 10.3389/fendo.2024.1433297 (PMC11250425; doi:10.3389/fendo.2024.1433297)
Supplement: Supplementary file 2 [file DataSheet_2.docx]

# S2. Rehabilitation exercises protocol

# Rehabilitation Overview

Regardless of whether the patients are in the face-to-face physical rehabilitation group or the tele-rehabilitation group, they are guided by principles specifically designed for HFpEF rehabilitation training, developed collaboratively by our hospital's cardiology and rehabilitation departments. The intervention will be administered once daily in the hospital and three days per week in the outpatient setting or in remote rehabilitation setting. Each session will consist of three stages: 1) warm-up (including seated core exercises, light walking, and stretching exercises), 2) rehabilitation/training (including four domains: endurance, mobility, strength, and balance), and 3) cool-down (stretching exercises). The rehabilitation/training portion of each session will include exercises tailored to the participant's performance.

# Initiation of the Rehabilitation Intervention and Initial Exercise Prescription

Participants received the multi-domain rehabilitation intervention in the hospital as soon as possible. Plans for starting or continuing the intervention in the outpatient setting or remote rehabilitation will be established before hospital discharge, with a schedule to commence within the first week post-discharge.

The initial rehabilitation exercise prescription will be customized for each participant based on their functional performance in each of the four domains. These domains include: strength, assessed by the ability to rise from a chair without hand support; balance, evaluated by standing ability; endurance, measured by continuous walking; and mobility, determined by gait speed.

# Modality of Exercise

1. Balance rehabilitation

Balance rehabilitation will include both static and dynamic exercises. For static balance, exercises will progress by holding increasingly narrow bases of support, starting with feet together, then moving to semi-tandem stance, tandem stance, and finally single-leg stance, initially with eyes open and then progressing to eyes closed.

For dynamic balance, participants will be challenged to reach further beyond their base of support during activities that progress from seated to standing. Dynamic balance will also be integrated into functional strengthening exercises, such as step and reach.

Close supervision is essential. In the outpatient setting, a PT will supervise, using tools like gait belts. For home exercises, the PT will send the necessary equipment beforehand and ensure the environment is safe for exercises, including the installation of handrails if needed. Patients will also be encouraged to have family members assist them to ensure safety.

(2) Aerobic exercise for endurance rehabilitation

Large muscle mass endurance exercises (walking or cycling) are recommended for 30 to 45 minutes, three days per week, at a moderate to high intensity to improve peak oxygen uptake (VO_2peak_), with an initial goal of 5-10 minutes total duration. The duration of aerobic exercise is adjusted by the physical therapist (PT) based on the patient's performance in the endurance domain.

Endurance training intensity can be prescribed based on maximal heart rate reserve (HRR), percentage of VO_2peak_ or rating of perceived exertion (RPE). Initially, the training intensity during the first few sessions is set at 40-50% of VO_2peak_ and should progressively increase to 70-80% of VO_2peak_ over several weeks as training adaptations and improved exercise tolerance occur. If peak heart rate (HR) is measured, the recommended training intensity is 40-70% of HRR, calculated as HRR = 40% to 70% (peak HR ‒ resting HR) + resting HR. In cases where VO_2peak_ or peak HR measurements are either not obtained, unattainable, or unreliable (e.g., due to β-blockade), the training intensity can be prescribed using the Borg Rating of Perceived Exertion (RPE) scale, targeting a range of 10-14 out of 20.

(3) Resistance training for strengthening rehabilitation

Resistance training is an effective method for enhancing both the quantity and quality of skeletal muscle.

To improve muscular endurance, lower intensity resistance exercises (30-40% of 1-RM, 10-25 repetitions) for both upper and lower extremities should be performed 2-3 days per week. For increasing muscular strength, higher intensity training (40-60% of 1-RM, 8-15 repetitions) should be conducted 2-3 days per week. The physical therapist (PT) will tailor the combination of these exercises based on the patient’s condition.

To ensure patient safety and appropriate muscular strength progression, it is important to start with lower intensity exercises and gradually increase the intensity over time. This approach helps prevent skeletal muscle injury and maximizes muscle adaptations.

(4) Mobility rehabilitation

To improve functional mobility (improve gait speeds and impaired dynamic balance), certain exercises can be combined with balance activities. These exercises will include dynamic start and stop movements and changing direction while walking, addressing both balance and mobility simultaneously. As participants' strength, endurance, and balance improve, their ability to perform these exercises will also enhance. Close supervision and guarding will be provided to prevent injuries and falls. If the home-based environment cannot ensure safety, this portion of the exercises will be minimized.
